# Advances in Cellular Therapy for the Treatment of Thyroid Cancer

**DOI:** 10.1155/2010/179491

**Published:** 2010-06-29

**Authors:** Claudia Papewalis, Margret Ehlers, Matthias Schott

**Affiliations:** Endocrine Cancer Center, Department of Endocrinology, Diabetes, and Rheumatology, University Hospital Duesseldorf, Moorenstr.5, 40225 Duesseldorf, Germany

## Abstract

Up to now, there are no curative therapies available for the subset of metastasized undifferentiated/anaplastic thyroid carcinomas. This review describes the possible use of immunocompetent cells which may help to restore the antitumor immune recognition for treating an existing tumor or preventing its recurrence. The most prominent experimental strategy is the use of dendritic cells (DCs) which are highly potent in presenting tumor antigens. Activated DCs subsequently migrate to draining lymph nodes where they present antigens to naïve lymphocytes and induce cytotoxic T cells (CTL). Alternatively to DC therapy, adoptive cell transfer may be performed by either using natural killer cells or ex vivo maturated CTLs. Within this review article we will focus on recent advances in the understanding of anti-tumor immune responses, for example, in thyroid carcinomas including the advances which have been made for the identification of potential tumor antigens in thyroid malignancies.

## 1. Introduction

The most frequently occurring forms of thyroid cancer have a good prognosis. The neoplasms originate from two distinct endocrine cell types of the thyroid gland: thyroid hormone producing follicular epithelial cells and calcitonin-producing parafollicular cells (C-cells). The most prevalent thyroid cancer is that of papillary origin (PTC; about 60–80%) it differs from follicular (FTC; 15–25%) and anaplastic carcinomas (ATC; 2–5%) but all are derived also from follicular epithelial cells. Only about 3–10% of the thyroid tumors derive from parafollicular C-cells. These tumors are termed as medullary thyroid carcinoma (MTC) [1, 2].

The good prognosis of PTC and FTC is originally attributed to the steady growth rate and the competence of taking up iodide. This leads to the potential classical therapeutic option of radioiodide (131I-) treatment after a surgical revision of the tumor mass [3]. The varieties of the different tumors of follicular origin reveal in their distinct morphology, their kind of metastatic spreading, and their molecular alterations leading to different pathways involved in the boost of cell proliferation [4]. 

The carcinoma bearing the worst prognosis is ATC which nearly almost derive *de novo* or rarely from pre-existing PTC or FTC from which it may dedifferentiate sometimes revealing transitional stages [5, 6] and, however, may develop into highly malignant ATC with low survival rates. ATC have a high mitotic rate with both hematological and lymphovascular invasions and do not retain any of the biological features of the original follicular cells, such as uptake of iodine and synthesis of thyroglobulin [7]. Due to their molecular alterations and biological inoperable feature these tumors are inaccessible to classical treatment options such as radio- and chemotherapy [8]. New drugs, such as fosbretabulin [9], bortezomib, and TNF-related apoptosis induced ligand (TRAIL) [10] are now introduced and trialed in clinical labs and human clinical studies. 

Beside, MTC might also have a good prognosis when it is restricted to the thyroid gland itself [11]. In case of distant metastases moieties of the patients develop a rapidly progressing disease leading to death. Clinical management of this disease is possible only by monitoring the location and expansion of the tumor mass and metastases followed by an extensive surgery and by observing antigens like calcitonin or carcinoembryonic antigen during the follow up [12]. 

Especially for the noncurative thyroid tumors such as ATC and all invasive and metastasized PTC and FTC it is of substantial interest to develop new approaches for the treatment of these cancers. Here, we can learn from immunological interventions which have already been applied to patients with other nonendocrine and endocrine cancers including MTC.

## 2. Advances in Cellular Therapy

An alternative approach for the treatment of cancers may represent the use of cell vaccines aiming to activate the immune system. Antitumor immunity is coordinated by both innate and adaptive immunity, and mainly mediated by cytotoxic T cells (CTLs), natural killer (NK), and natural killer T (NKT) cells. The induction and coordination within this context is arranged by dendritic cells (DCs) [13, 14]. DCs are highly potent antigen-presenting cells with the ability of taking up and processing tumor antigens in the peripheral blood and tissues. They subsequently migrate to the draining lymph nodes to present antigens to naïve T lymphocytes and induce a cellular immune response by direct priming of CD8+ CTLs and a cross-presentation involving CD4+ helper cells. Moreover, DCs are also important in inducing humoral immunity as explained by their capacity to activate naïve and memory B cells [15] and NK [16] as well as NKT cells [17]. Thus, DCs can modulate the whole immune repertoire they represent an excellent tool for treating an existing tumor or preventing its recurrence.

*In vivo*, two main pathways of DC differentiation have been described depending on their cell lineage and the tissue were they are activated [18]. Under *in vitro* conditions, myeloid DCs may be generated from monocytes by activating with granulocyte/macrophage colony-stimulating factor (GM-CSF). The monocytes which encounter interleukin-4 (IL-4) become DCs known as IL-4-DCs [19–21]. On the other hand, monocytes differentiate into different DC subsets after costimulation with other cytokines such as IFN-*α*, TNF or IL-15 [22–28]. One essential precondition for the success is the priming of DC with special tumor associated antigens (TAA). These TAA must bear high immunogenic properties against the background of HLA restriction. In this context, the broadest experience has been gained for the therapy of malignant melanoma [29–33] as well as in renal cell carcinoma [34–38]. Even though only limited success has been achieved from the clinical point of view, DC vaccinations are superior compared to other antitumor vaccination strategies [39]. Importantly, DC vaccination strategies have steadily been improved by the number of immunocompetent tumor antigens identified thus far [40–43], even in endocrine malignacies [44]. One way of assessing tumor antigens is based on the use of computer-based algorithm software namely SYFPEITHI or BIMAS [45–48] which helps to predict immunocompetent tumor epitopes. Examples of classical tumor epitopes are those of MAGE, GAGE, and NY-ISO1 in malignant melanoma [49–55]. 

Alternatively to DC therapy, new strategies were introduced using antigen-specific CTLs or potent NK cells to burst the immune tolerance. Adoptive T-cell immunotherapy is mainly performed by the generation of large numbers of antigen-specific CTLs. But both tumor-specific CD4+ helper 1 (Th1) cells and cytotoxic CD8+ T cells might be generated *in vitro* and administered directly to patients [56, 57]. Maturation of specific lymphocytes were performed by *ex vivo* exposure to cytokines, HLA-restricted antigen epitopes, and either *in vitro* generated DCs [57, 58] or with CD3 and/or CD28-specific mAb [58, 59].

On the other hand, NK cells are the major representatives of the innate immune system that is regulated by positive and negative mechanisms. NK cells interact with tumor cells through activating receptors of the immunoglobulin superfamily (NKp30, NKp44, NKp46, NKG2D) and the lectin-like type II transmembrane proteins exhibiting a C-type lectin domain (CD94, CD161), and on the other hand, through inhibitory receptors (KIR, e.g., NKG2A) which primarily bind to MHC class I antigens on target cells [60, 61]. Moreover, NK cells express CD16 which serves as a receptor for antibodies to home on target cells performing the antibody dependent cellular cytotoxicity [62, 63]. 

Within the last decade a multitude of different studies and clinical trails have been performed using cell therapies [64–67]. Cancer immunotherapy using DC or adoptive CTLs has much promise because malignant cells can be affected by the immune system without damaging healthy tissue and without dangerous side effects. Nevertheless, careful monitoring of the elicited T-cell response and quality assurance is mandatory to establish a rationale for specific immunotherapy and to bring it from bench to bedside [68, 69]. In any case, the identification of tumor cell specific antigens is crucial for establishing clinically effective tumor immunotherapies and monitoring the induced immune response, including quantification of antigen-specific CTLs. Other approaches might use NK or NK-T cells, respectively, however with less experiments compared to DC vaccinations [70, 71].

## 3. Experience in Cellular Therapies for Medullary Thyroid Carcinoma

The idea of using polypeptide hormones as tumor antigens for cancer therapy resulted from the observation in autoimmunity, where responses to self tissue antigens led to tissue damage. The most intensively investigated autoimmune disease is T1DM, which is characterized by infiltration of pancreatic islets by self-reactive lymphocytes, leading to destruction of insulin-secreting *β*-cells. Insulin itself is probably the most important autoantigen described thus far [72]. This is supported by the fact that many reactive T cells invading pancreatic islets are specific for immune insulin epitopes and are capable of adoptively transferring diabetes in non-obese diabetic (NOD) mice [73, 74]. 

This violability of the immune system leads to the option to reconstitute immunity by antitumor immunotherapy. One goal was scored by the work of Bradwell and Harvey identifying the use of polypeptide hormones as tumor antigens [75]. They were first to apply a combination of synthetic human and bovine parathyroid hormone (PTH) peptides for vaccination of one patient with metastatic parathyroid cancer. They demonstrated increased PTH-specific antibody titers, resulting in a notable decrease in serum calcium levels and a relief of clinical symptoms. Nonetheless, no association with reduced tumor mass was observed. Thereafter, Betea et al. [76] performed an immunization trial with bovine and human PTH fragments and with intact full-length human PTH. The effect of this treatment was a specific antibody production to all PTH fragments, resulting in largely diminished PTH and serum calcium levels. Most importantly, they observed a remarkable decrease in tumors of pulmonary metastases, indicating a PTH specific cytotoxic immune response.

Based on this knowledge, the polypeptide hormone calcitonin has been proposed as tumor antigen for immunotherapy in MTC. Since then, several vaccination trials have been performed in murine models and as well in man. Vaccination studies with CT-loaded DCs were performed in a transgenic mouse model for MTC mice displaying the identical mutation (substitution of Cys for Arg) within the *RET *protooncogene at codon 634 as most patients with multiple endocrine neoplasia type 2A [77, 78]. As in patients with hereditary MTC, Ret/Cal mice develop diffuse C cell hyperplasia and MTC with increased serum CT levels [77]. Depending on the CT epitopes used epitope specific CD8+ CTLs were visualized via tetramer analyses and by functional lysis assays. These results were accompanied by a largely diminished tumor outgrowth [79, 80].

In humans, several studies used full-length CT for priming DCs [81, 82]. Importantly, in one patient, a remarkable transient regression of pulmonary and liver metastases was seen. Detailed *in vitro* analyses revealed a CT-specific T-cell reactivity, which was Th1-driven, in some patients, as determined by a large increase in IFN-*γ* production [83]. Thereafter, a new protocol with interferon-*α* generated DCs with direct tumor lysis activity was performed [84]. These cells were also loaded with full-length CT [85]. After a long-term follow-up of more than 48 months, two of five MTC patients showed stable disease with changes in tumor size and tumor marker of less than 25%. This is important because it shows a direct connection between induction of cytotoxic immunity and clinical response [85].

## 4. Potential Tumor Antigens in Poorly Differentiated Thyroid Carcinomas

Tumor-associated antigens (TAA) are surface-associated molecules such as receptors, transmembrane proteins or secreted/membrane-attached peptides that are mostly cancer specific, often overexpressed and recognized by the immune system [120]. Therefore, identifying specific TAA is of key importance for developing new options for immunotherapy for incurable cancers. Up to now, however, no single TAA for primary thyroid carcinomas have been proven but there are a couple of candidates which might have the potential to become one.

Potential tumor antigens which might represent a distinct tumor association can be divided into the groups of classical cancer testes antigens, specific receptors, functional-associated proteins, and metastases-associated proteins. Find an assembly of potential TAA and of already performed experiments in Table 1.

The most prominent tumor antigens are certainly the *cancer testes antigens*, which have already been identified in many malignancies and which have intensively used in the context of immunotherapy [31–33, 40–42, 50–52]. These antigens belong to a gene family which has been reported to be expressed in tumor cells but not in normal tissues aside from the testicular germ cells where the absence of MHC class-I molecules protect the cells from testicular autoimmunity, as the antigenic peptides are not be displayed at the cell surface [121]. This makes these TAA so attractive for immunotherapy since no side effects are expected. In PTC and FTC the cancer testis genes *MAGE* and *GAGE* were identified in human thyroid carcinomas [86–88]. For instance, MAGE-3 is detectable in 29% of follicular tumor tissue and in 80% in papillary thyroid carcinomas. This observation explores the possibility of specific immunotherapy using these TAA for vaccination trails. 

Another group of potential tumor antigens represent the large group of *receptors*. The main player are the IGF-I receptor in thyroid carcinomas [89, 90, 122] and the receptor tyrosine kinases as EGF-R, PDGF-R *α* & *β*, VEGF-R 1&2, c-KIT especially in FTC and some ATC [91, 93, 94]. Whether these receptors are likely to be used as tumor antigens has still to be proven. IGF-I is known to have significant effects on cell proliferation and differentiation, it is a potent mitogen, a powerful inhibitor of programmed cell death, and has a well-established role in the transformation of normal to malignant cells. So, especially the overexpression of the IGF-I receptor might have a possible target function while its presence is important for the development of a malignant phenotype [89, 123]. Up to now, however, only antibody-based therapies were performed [90, 124].

Other receptors untypically expressed in thyroid carcinomas might also be considered. For example, CD10 a common antigen for acute lymphoblastic leukemia has been found to be useful in the differential diagnosis of malignancy. Moreover, it has been shown to be expressed in a group of PTC as well as in papillary microcarcinomas [95] and in some FTC [96]. Several reports implicated the chemokine receptor CXCR4 in thyroid tumor aggressiveness. The target for CXCR4 is the chemokine CXCL12/SDF-1 *α* & *β* involved in both embryonic and tumor angiogenesis [97]. This receptor might be also a potent target for a direct CTL offense.

In 2007, characteristic biomarkers were described for PTC lymph node metastasis [98]. By real-time reverse transcription-PCR and immunohistochemistry three genes were discovered consistently overexpressed in lymph node metastasis. Especially, LIM (kinase) domain containing 2 (LIMD2) and the protein tyrosine phosphatase receptor type C (PTPRC also known as CD45) were significantly different expressed in tumor samples versus metastatic samples. Additionally, lymphotoxin beta (LTB), a type II membrane anchored protein of the TNF family had borderline significance. Since there are no antibodies for LIMD2, only PTPRC and LTB could be tested by immunohistochemistry. All samples tested, showed both proteins in metastases and little or no expression in primary tumor. 

Moreover, there are the so-called *functional-associated antigens*. PTC and FTC derive from thyroid epithelial cells resulting in a large expression of thyreoglobulin which also represent a classical tumor marker [99, 100, 125, 126]. Whether thyroglobulin also represents a tumor antigen needs, however, to be proven.

Beside, a couple of other antigens have recently been described for thyroid carcinomas. One of those is a biomarker for the malignancy status of PTC and FTC. The human mesothelial cell marker 1 (HBME-1) have originally been decribed in mesotheliomas. Meanwhile, it is known that it is expressed in 90 to 100% of the carcinomas with a strong membrane staining [102, 103]. Additionally, several other antigens are on debate like human type I cytokeratin 19 (CK19), galectin-7, and probably galectin-3 which are overexpressed in papillary and follicular malignancies [102, 127, 128]. CK19 is an acidic protein of 40 kDa that is part of the cytoskeleton of epithelial cells and is highly expressed by differentiated thyroid carcinomas, mainly of the papillary subtype. The soluble fragments of CK19 can already be measured by immunometric assays [129]. Galectins are a structurally related family of lectin proteins that bind specifically to beta-galactoside in a calcium-independent manner; originally they are cytosolic proteins involved in growth regulation and internal processes such as pre-mRNA splicing [130] but they are able to translocate into vesicles due to participate in cell-cell and cell-matrix adhesion [130, 131]. Moreover, elevated levels of fibronectin-1 might be a good target due to its alternative splicing during tumorigenic process which leads to different isoforms of extracellular domains or connecting segments [101, 102]. Likewise, two other TAA which have a broad expression pattern in many types of human malignancies are now described in thyroid carcinomas as well. One is survivin which is overexpressed in poorly differentiated thyroid cancers inducing antiapoptotic processes [104] and the telomerase reverse transcriptase (TERT), that is, concomitant in cancer cells responsible for the stabilization of the telomeres receiving an immortalization of the respective cells [105–108]. Both antigens where already used as targets for several vaccination studies in melanomas and breast cancer [132–137].

Finally, there is the group of *metastases-associated proteins.* The growth of a neoplasm and its ability to metastasize is a multistep process dependent on angiogenesis and immunological reactions of the organism. In this process, adhesive factors like soluble intercellular adhesion molecules (sICAM-1) and vascular cellular adhesion molecules (sVCAM-1) are involved. The serum of peripheral blood of patients with thyroid cancer before surgery revealed these factors in a significant higher concentration compared to controls [138]. Since these soluble factors are egested by the tumor itself and might also be associated to the membrane exhibiting a potent tumor antigen. 

Aside, several other factors are described being involved in invasion and metastasis. One main component is the group of matrix metalloproteinases (MMPs) since they disrupt extracellular matrix proteins. An increase of circulating MMP-2 [109, 110, 139, 140], MMP-7 [110, 111], and MMP-9 [110, 112, 141] was affirmed manifold. In this context, CD147 one of the molecules involved in regulating the expression MMP-2 was described to be expressed more frequently in PTC and ATC patients and is associated with their clinicopathologic features [109]. Additionally, the urokinase-type plasminogen activator (u-PA) and its specific receptor (u-PA-R) are involved in the disruption of the extracellular matrix which relies on the activation of plasminogen and an interaction with MMPs [112, 113]. One report advised on an antigen, namely, fascin which is markedly upregulated in more than 60% PTC. It is displaying an actin-bundling protein, however, associated with high-grade extensive invasion [114].

## 5. Special Situation in Anaplastic Thyroid Carcinoma

In ATC, a multitude of molecular alterations are found [142, 143] but only a small number of potential antigens have been described. Nonetheless, some potential antigens discovered for FTC and PTC are also found in ATC although molecular variances may also lead to a downregulation of those. The most prominent loss is described for c-Kit which was monitored to be absent in most ATC [144, 145]. 

Two additional proteins were recently found in ATC. One is called autotaxin which has a nucleotide pyrophosphatase/phosphodiesterase and lysophospholipase D activity. It is usually secreted but also membrane-associated and is a highly bioactive enhancer for motility of thyroid tumors [115, 116]. Beside, expression of CD133 displaying a hematopoietic stem cells antigen was described in tumor derived cells lines [117, 118].

## 6. Future Perspectives

The research for cellular cancer therapy has bred some promising approaches but until now no single vaccination regimen tested is indicated as a standard anticancer therapy. In order to circumvent the escape of thyroid tumor cells under T-cell pressure, polyvalent vaccination strategies may help to overcome this situation. This goal can be achieved by either loading DCs with a pool of peptide antigens which might be individually identified as TAAs or by adoptive CTL or NK/NK-T cell transfer. The major drawback in many human malignancies including thyroid tumors is, however, the lack of established tumor antigens which, in addition, have already been applied in clinical context. As mentioned above, a multitude of proteins and receptors have been described to be overexpressed in a certain percentage of these thyroid tumors. Whether some of those are true TAAs recognized from cells of the adaptive immune system is still elusive and needs to be clarified. Not till then, they could be used in clinical trials in humans. Nonetheless, it is necessary to search for other potential tumor antigens. Within this context, novel technologies, that is, high-throughput gene microarray, should further be implemented in order to identify new antigens.

Another way of improving present treatment concepts is to use a combination therapy by which tumor cells are selectively affected and the tumor escape mechanisms are accessory blocked or decreased [146]. In this context, conventional chemotherapy has already been supported by a combination with DC vaccination showing some clinical benefits in nonendocrine tumors [147–149]. In endocrine (e.g., thyroid) tumors such data does, however, not exist. More relevant, however, might be a combination of cellular immunotherapy and tyrosine kinase inhibitors (TKIs) affecting target-specific agents. In thyroid malignancies different TKIs particularly sorafenib, motesanib, vatalanib, and so forth, have already been applied in clinical studies [150–152]. Although TKIs have been described to deplete immunoregulatory, for example, regulatory T cells they also have an effect on all T cells and DCs [153, 154] but interestingly not on NK cells [155]. On the other hand, TKIs' affect DCs to activate NK cells [156–158]. The depletion of T cells by TKIs also resulted in a reconstitution with a predominant expansion of antigen-specific T cells [159] and the higher binding capacity of CTLs to MHC presenting antigens [160]. So, the combination of cellular therapies with targeted molecules including TKIs hold promise for successful cancer therapies in the future.

## Figures and Tables

**Table 1:** List of possible thyroid tumor associated antigens.

		Methods			
Tumor	Potential TAA	Biochemical		in vivo	
		RT-PCR	IHC	Animal model	Clinical trial
	*Cancer antigens*				
PTC	MAGE	× [86, 87]	× [86–88]		
FTC	GAGE	× [87]	× [87]		
	*Receptors*				
	IGF-I R	× [89]	× [89, 90]		
	EGF-R		× [91, 92]		
	PDGF-R	× [93]			
	VEGF-R	× [93, 94]	× [94]		
	c-KIT	× [93]			
	CD10		× [95, 96]		
	CXCR4		× [97]		
	LIMD2	× [98]	× [98]		
	CD45	× [98]	× [98]		
	LTB	× [98]	× [98]		
	*Functional associated*				
	Thyreoglobulin	× [99–101]			
	HBME-1		× [102, 103]		
	CK19	× [101]	× [102]		
	Galectins		× [103]		
	Fibronectin-1	× [101]	× [102]		
	Survivin		× [104]		
	TERT	× [105–107]	× [108]		
	*Metastases associated*				
	MMPs		× [109–111]		
	CD147		× [109]		
	u-PA/u-PA-R	× [112, 113]			
	Fascin		× [114]		

ATC	Autotoxin	× [115, 116]			
	CD133	× [117]	× [117, 118]		

MTC	CT			× [84]	× [81, 82, 85]
	PPCT			× [80, 85]	
	CEA				× [81, 82]
	NY-ESO-1	× [119]			

ATC: anaplastic thyroid cancer; FTC: follicular thyroid cancer; MTC: medullary thyroid cancer; PTC: papillary thyroid cancer.

MAGE/GAGE: melanoma antigens, also known as cancer testis antigen; IGF-I R: insulin growth factor-receptor; EGF-R: epidermal growth factor-receptor; PDGF-R: plateled-derived growth factor-receptor; VEGF-R: vascular endothelial growth factor-receptor; c-KIT: hematopoietic stem cell receptor; CD10: common acute lymphocytic leukemia antigen; CXCR4: stromal cell-derived factor 1 receptor; LIMD2: LIM kinase containing receptor-2; CD45: protein tyrosine phosphatase receptor type C; LTB: lymphotoxin beta membrane anchored protein; HBME-1: human mesothelial cell marker 1; CK19: cytokeratin 19; TERT: telomerase reverse transcriptase; MMPs: matrix metalloproteinases; CD147: basigin, a subunit of the monocarboxylate transporter; u-PA and u-PA-R: urokinase-type plasminogen activator and its receptor; CD133: hematopoietic stem cells antigen; CT: calcitonin; PPCal: preprocalcitonin; CEA: carcinoembryonic antigen; NY-ESO-1: a cancer testis antigen.

RT-PCR: real-time PCR; IHC: immunohistochemistry.
